# Phytochemical Profile of *Opuntia ficus-indica* (L.) Mill Fruits (cv. ‘Orito’) Stored at Different Conditions

**DOI:** 10.3390/foods11020160

**Published:** 2022-01-08

**Authors:** Francisca Hernández, Lucía Andreu-Coll, Andreia Bento-Silva, Ana Teresa Serra, Pedro Mena, Pilar Legua, Maria Rosario Bronze

**Affiliations:** 1 Grupo de Investigación en Fruticultura y Técnicas de Producción, Centro de Investigación e Innovación Agroalimentaria y Agroambiental (CIAGRO-UMH), Miguel Hernández University, Carretera de Beniel, km 15 3.2, 03312 Orihuela, Alicante, Spain; lucia.andreu1@gmail.com (L.A.-C.); p.legua@umh.es (P.L.); 2 Instituto de Investigação do Medicamento, Faculdade de Farmácia, Universidade de Lisboa, Avenida Prof. Gama Pinto, 1649-003 Lisboa, Portugal; abentosilva@ff.ulisboa.pt (A.B.-S.); mbronze@ibet.pt (M.R.B.); 3 Departamento de Ciências Farmacêuticas e do Medicamento, Faculdade de Farmácia, Universidade de Lisboa, Av. das Forças Armadas, 1649-003 Lisboa, Portugal; 4 Laboratory of Phytochemicals in Physiology, Department of Food Science and Drugs, University of Parma, Medical School, Building C, Via Volturno, 39, 43125 Parma, Italy; tserra@ibet.pt; 5 Instituto de Biologia Experimental e Tecnológica, Avenida da República, Estação Agronómica Nacional, 2780-157 Oeiras, Portugal; 6 Instituto de Tecnologia Química e Biológica, Universidade Nova de Lisboa, Avenida da República, Estação Agronómica Nacional, 2780-157 Oeiras, Portugal; pedromiguel.menaparreno@unipr.it

**Keywords:** prickly pear, HPLC-MS, betalains, phenolic acid, lignans, antioxidants

## Abstract

This research analyzed the phytochemical profile of prickly pear fruits from ‘Orito’ cultivar stored under cold conditions (2 °C, 85–90% RH) and shelf-life conditions at room temperature (stored at 20 °C for three days after cold storage) for 28 days, mimicking the product life cycle. A total of 18 compounds were identified and quantitated through HPLC-DAD-MS/MS (High-Performance Liquid Chromatographic -Diode Array Detector- Mass Spectrometry) analyses. Phenolic acids such as eucomic acid and betalains such as indicaxanthin were the predominant chemical families, and piscidic acid was the most abundant compound. During cold storage, the content of eucomic acid isomer/derivative and syringaresinol increased, and citric acid decreased, which could be caused by the cold activation of the phenylalanine ammonia-lyase (PAL) and polyphenol oxidase (PPO) enzymes. However, no significant differences were found in the content of these compounds during shelf-life storage. These results showed that ‘Orito’ fruit marketability would be possible up to 28 days after harvesting, retaining its profile, which is rich in bioactive compounds.

## 1. Introduction

Prickly pear is a sweet flavory fleshy berry with thick peel and many seeds, varying in size, shape, and color. This fruit has excellent nutritional properties, is low in calories and high in bioactive compounds, such as betalains, phenolics and vitamins, which show antioxidant activity and are related with health benefits and the prevention of some chronic diseases, due to their anti-inflammatory, antidiabetic, neuroprotective and antiproliferative activities, among others [1,2,3]. 

Besides its consumption as a fresh fruit product, prickly pear presents a wide range of applications, including animal feeding, developing of processed products such as juices and jams, and production of bioethanol and biogas. Due to the high concentration of bioactive compounds, especially (poly)phenolic compounds, prickly pear can be used in the nutraceutical, pharmaceutic, and cosmetics industries [4,5,6]. The (poly)phenolic composition of prickly pear fruits and derived products has been well described, and includes phenolic acids, flavonoids, and lignans, among others [2,7]. In addition, fruits present high amounts of betalains [2].

Postharvest conservation affects quality characteristics of fruit and vegetables because they continue their metabolic changes after harvesting. In non-climacteric fruits, these changes can be undesirable and cause quality losses [8,9]. For prickly pear fruit, chilling injuries, microbial growth, loss of firmness and weight loss are the major deterioration factors that affect this fruit after harvesting and during postharvest conservation, its quality depending pretty much on handling and storage conditions [8,10].

Although prickly pear was classified as a non-climacteric fruit, classification of climacteric and non-climacteric fruit is not absolute, and genotypes and cultivars of some species can show both patterns [11,12]. ‘Orito’ fruit, the commercial Spanish cultivar studied in this work, showed a suppressed climacteric pattern in ethylene production and respiration rate similar to some cultivars of plum, which showed no increase in respiration rate or in ethylene production associated with ripening [13,14,15]. In a previous work we demonstrated that ‘Orito’ fruit maintained its quality parameters in desirable values up to 28 days, both in cold conditions (2 °C, 85–90% relative humidity, RH) and during shelf-life storage, whereas the total phenolic content increased during the shelf life conditions [13]. However, to this point in time there are no studies evaluating the (poly)phenolic composition of prickly pear fruits during storage. The aim of this work was to evaluate the impact of storage conditions and shelf-life on the bioactive compound profile of ‘Orito’ fruit, with a particular focus on the (poly)phenolic fraction. The results obtained will contribute to select the best shelf-life and storage conditions to improve marketability of the fruits and to develop further strategies focused on the valorization of the phytochemical profile of this species. 

## 2. Materials and Methods

### 2.1. Plant Material and Sample Processing 

Fruits of a commercial cultivar of *Opuntia ficus-indica* (L.) Mill., called ‘Orito’, were used for this study. The fruits were hand-harvested in mid-August 2017 in a commercial farm (38°23′30.7″ N, 0°40′13.0″ W, Orito, Alicante, Spain). The fruit was collected at the commercial ripening stage and was carried in cold to the laboratory for sample preparing and further analyses. At the laboratory, fruits were brushed to remove the spines. Next, 540 fruits were chosen based on homogeneous size, color, and by the absence of visual defects. These fruits were indiscriminately divided into 27 lots of 20 fruit, each being a biological replicate. 

Three lots were used for evaluating the fruit properties at. The rest of the lots were stored in a refrigeration chamber at 2 °C and 85–90% RH (cold conservation). Of these, three lots were reserved for evaluation at 7, 14, 21, and 28 days after harvest. Besides, the other three lots for each time point were taken and disposed of at 20 °C for three days to study shelf life (SL). The pulp of the fruits was cut into small pieces to achieve a uniform sample of each biological replicate and were directly frozen at −80 °C. After freezing, samples were freeze-dried in an Alpha 2.4 freeze drier (Christ Alpha 2.4; Braum Biotech, Osterode am Harz, Germany) for 24 h at a reduced pressure of 0.220 mbar. The temperature in the drying chamber was −25 °C, while the heating plated reached 15 °C. At the end of the freeze-drying, the samples were powdered and packed in vacuum until analysis. The information about samples (code and description) is summarized in Table 1.

### 2.2. Extraction of Phytochemical Compounds 

Phytochemicals were extracted following the protocol of Mena et al. [2]. Briefly, 200 mg of freeze-dried powder were mixed with 1 mL of 80% aqueous methanol acidified with formic acid (1%). This mixture was sonicated for 25 min, and the mixture was then centrifuged at 10,480× *g* for 5 min at room temperature. After supernatant collection, two additional extractions were executed for each sample with an additional 0.5 mL of the extraction solvent, as described above. All three supernatants were pooled and filtered through a 0.45 µm Millipore filter (Billerica, MA, USA) before HPLC-MS analysis. Final extracts presented a concentration of 0.1 g dw mL^−1^. Each sample was extracted in triplicate. 

### 2.3. HPLC-DAD (High-Performance Liquid Chromatographic-Diode Array Detector) Analysis

The extracts were analysed in a HPLC Dionex Ultimate 3000 equipped with a C-18 LiChrospher^®^ 100 RP-18 (5 μm) column (250 × 4.0 mm) (Sigma-Aldrich, San Luis, MO, USA) operating at 35 °C, coupled to a DAD-3000 detector (Thermo Scientific, Massachusetts, USA). The mobile phase consisted of water-formic acid (0.5% *v*/*v*) (eluent A) and acetonitrile (90%) + formic acid (0.5%) + water (eluent B) at a flow rate of 0.3 mL/min with an injection volume of 20 μL. The gradient used is summarized in ‘Supplementary Materials Table S1’ section.

### 2.4. HPLC-DAD-MS/MS Analysis

Samples were also analysed by an HPLC-DAD-MS/MS system: a Waters Alliance 2695 (Waters^®^, Dublin, Ireland) separation module with an autosampler (20 μL injection volume), a quaternary pump and a solvent degasser, coupled to a Photodiode Array Detector Waters 996 PDA (Waters, Dublin, Ireland) scanning wavelength absorption between 210 and 600 nm. A LiChrospher^®^ 100 RP-18 5 μm column at 35 °C (stabilized by a column oven) was used. Tandem mass spectrometry (MS/MS) detection was carried out with a Micromass^®^ Quattro Micro triple quadrupole (Waters, Dublin, Ireland), using an electrospray ionization source in both positive (ESI+) and negative (ESI-) modes. A full scan mode (*m*/*z*: 60–1100) record was applied for the mass spectra of the compounds separated by HPLC, using a collision energy of 20 eV. The HPLC gradient method and eluents are described in ‘Supplementary Materials Table S2’ section. The MS/MS conditions, as source temperature, capillary and source voltages have been previously described by Katsinas et al. [16]. For data acquisition and processing, MassLynx^®^ 4.1 software (Waters, Dublin, Ireland) was used.

### 2.5. Statistical Analyses 

One-way analysis of variance (ANOVA) and multiple-range tests were used for samples comparison. The data were compared throughout cold storage and under shelf life conditions independently (from day 0 to day 28) and each day was independently compared under cold and shelf life conditions. The method used to discriminate among the means (Multiple Range Test) was the Tukey’s least significant difference procedure. Significance was defined at *p* ≤ 0.05. Statistical analysis was performed using XLSTAT software version 9 (Microsoft Corporation, Redmond, WA, USA) [17]. 

## 3. Results and Discussion 

The exhaustive analysis of *O. ficus-indica* fruit pulp phytochemical composition allowed the tentative identification of 18 compounds (Table 2). Taking into account the compounds identified in prickly pear fruit pulp, betalains (four compounds, namely 14,15 and 17), phenolic acids (nine compounds, of which 3–7 were phenylpyruvic acids and 8 and 13 were hydroxycinnamic acids), lignans (four compounds, 9–12) were the most relevant classes of phytochemicals. In addition, some other compounds such as organic acids (compounds 1 and 2), an amino acid (16) and a prenylflavonoid (compound 18) were detected. These compounds were identified based on their retention time, fragmentation patterns obtained from mass spectra and by comparing their mass spectral characteristics with the available literature. 

Piscidic acid (component 3) was the compound which showed the largest area (Table 3) and some authors reported this acid as the most abundant compound in prickly pear fruits [21]. This compound showed no significant changes during cold and shelf life storage, and neither did eucomic acid (component 5). Piscidic acid is a chelator of iron which shows strong antioxidant activity, and its presence is unusual in nature, being restricted to crassulacean acid metabolism (CAM) and succulent plants [21,22]. Although there are no data about the antioxidant properties of eucomic acid, Mata et al. [22] suggested that, due to their structure, it may be similar to that of piscidic acid.

No major changes were observed for most of the phytochemicals during the cold storage of ‘Orito’ fruit, while three-day shelf life after storage did not change the phytochemical profile of prickly pear fruits (Table 3). The content of eucomic acid isomer/derivative (7) and syringaresinol (12) increased, and the content of citric acid (2) decreased (Table 3). Compounds 7 and 12 increased their content starting on day seven of cold storage, and from this moment they remained stable until the end of cold storage. However, in these compounds no significant differences were found in the comparison of cold and shelf life in the same day of storage. Ferulic acid derivative (8) decreased its content after seven days of cold storage, increased by day 21, and then decreased slightly again at the end of cold storage. 

Compound 12, which content increased under cold storage, pertained to the family of lignans, a group of secondary metabolites recognized as phytoestrogens and present in a great variety of plants, including seeds and some botanical berries as prickly pear. They are polyphenolic compounds related with the phenylalanine metabolism and show antioxidant properties related to anti-inflammatory, antitumoral and antiviral effects, among others [25,26].

Phenolic acids, as compounds 3–8 and 13, are aromatic secondary plant metabolites and are also widely spread throughout vegetables. They are related to color, sensory qualities, nutritional and antioxidant properties. There is some evidence of their role in the inhibition of oxidative damage diseases such as coronary heart disease, stroke and cancers [27,28]. Most of them did not change from being stored, which agrees with the results of this study, in which only compounds 7 and 8 changed during cold storage, and all of them remained stable under shelf-life conditions. 

Betalains, as compounds 14, 15 and 17, are water-soluble pigments responsible for the red or yellow color of fruits and other botanical parts such as flowers and leaves of species belonging to the order of Caryophyllales, in which prickly pear is included. These compounds, which remained stable under cold and shelf-life conditions, also show antioxidant activity and may provide health benefits to consumers [29,30]. 

Betalains, phenolic acids and lignans are compounds related with the phenylalanine metabolism. Phenylalanine is an aromatic amino acid which is a precursor or secondary metabolite in plants, serving as a building block of many compounds essential to plant structure, reproduction, defense, and communication [31]. Phenylalanine ammonia-lyase (PAL) is an enzyme regulating the synthesis and accumulation of phenolic compounds in plants. This enzyme is stimulated by cold, thus, the variation of phenolic content in fruits during cold storage might be influenced by its activation. Some studies reported the increase of PAL activity during cold storage, in parallel to the increase in phenolic compounds in strawberry, artichokes and blueberries [32,33,34]. 

Citric acid (2) content decreased during cold storage. This decrease could be attributed to the effect of the enzyme polyphenol oxidase (PPO), which may affect the concentration of other organic acids and is stimulated by cold storage [35]. 

There were no detected chilling injuries in ‘Orito’ fruit as they were found in other cultivars such as ‘Copena-Torreoja’, as studied by Corrales-García et al. [10]. In this sense, their results [10] showed 100% injury from chilling after the first month of cold storage. Other cultivars studied by these authors showed chilling injuries after two or three months of cold storage. Although visually ‘Orito’ fruit did not show such chilling-injury signs as browning, either under cold nor shelf life conditions, PAL and PPO enzymes are related to physiological disorders during cold storage in some fruits such as apples, mandarin-fruits and plums [33,35,36]. More studies are required to evaluate the chilling-injury sensitivity of the ‘Orito’ cultivar after longer cold storage. 

As previously mentioned, all of the identified compounds remained stable during shelf-life storage. This behavior could be attributed to the non-stimulation of these enzymes under shelf-life conditions. For instance, PAL activity and concentration decreased when mandarin fruits were exposed to non-chilling temperatures [36]. 

## 4. Conclusions

Based on the results of this study, ‘Orito’ fruits maintained their phytochemical composition during three-day shelf-life storage after cold storage, while the content of some betalains, phenolic acids and lignans increased during cold storage. These results, along with a previous study [13], showed that the marketability of prickly pear fruit from the ‘Orito’ cultivar can be possible up to 28 days after harvesting. Further investigation is required to evaluate the changes on the (poly)phenolic and betalain profile of these cactus fruits under other postharvest conditions, such as modified atmosphere packaging. In addition, assessing the role of well-known enzymes in the metabolism of these phytochemicals may lead to mechanistic insights useful to better handling postharvest reactions in CAM fruits.

## Figures and Tables

**Table 1:** Description of samples of *O. ficus-indica* (L.) Mill analyzed in this study.

Code	Description
D0	Day 0 (harvest)
D7	seven days in cold conservation
D7SL	seven days in cold conservation + three days at room temperature to study shelf life
D14	14 days in cold conservation
D14SL	14 days in cold conservation + three days at room temperature to study shelf life
D21	21 days in cold conservation
D21SL	21 days in cold conservation + three days at room temperature to study shelf life
D28	28 days in cold conservation
D28SL	28 days in cold conservation + three days at room temperature to study shelf life

**Table 2:** Retention time (RT) and characteristic MS ions of phytochemical compounds identified in prickly pear fruits during cold and shelf life storage.

Id.	Compounds	RT (min)	Percursor Ion [M-H]^−^ or [M]^+^ (*m*/*z*)	λmax (nm)	Product Ions ^†^ (*m*/*z*)	References
1	*L*-Malic acid	8.10	133^†^	272	71	[2]
2	Citric acid	11.17	191	466, 270	111, 87, 85, 43, 41, 67, 57	[2]
3	Piscidic acid	19.20	255	466, 274	73, 107, 165, 58, 93, 133, 179	[2,18]
4	Dicaffeoylferulic acid isomer 1	26.08	517	278	187, 239	[19]
5	Eucomic acid	27.75	239	274	107, 133, 149, 177, 179, 87	[2,18,20]
6	Dicaffeoylferulic acid isomer 2	27.82	517	274	239, 198	[19]
7	Eucomic acid isomer/derivative	29.89	239	268	179, 91	
8	Ferulic acid derivative	30.45	517	268	175, 193, 235	[2]
9	Guaiacyl(t8-*O*-4)guaiacyl-hexoside isomer 1	30.45	537	268	195, 324, 165	[2]
10	Guaiacyl(t8-*O*-4)guaiacyl-hexoside isomer 2	32.26	537	273	375	[2]
11	Secoisolariciresinol-hexoside	35.19	523	276	361, 447	[2]
12	Syringaresinol	46.80	417	275	181, 387, 166, 123	[2]
13	Feruloyl derivative	57.98	562	321,285	-	[2]
14	Betaxanthin- proline (indicaxanthin) isomer 1	19.85	309	480	106, 70, 263, 217	[21,22]
15	Betaxanthin-proline (indicaxanthin) isomer 2	20.51	309	480	106, 70, 263, 217	[21,22]
16	Tryptophan	31.61	205	278	118, 146, 132, 170	[23]
17	Phenylalanine-betaxanthin	37.28	359	480	315, 313, 131	[22]
18	Prenylnaringenin (trihydroxy-8-prenylflavanone)	38.27	341	275	137, 175, 251, 119, 311	[24]

^†^ Compounds 1–13 were identified in negative ionization mode, while compounds 14–18 were detected in positive mode. RT, retention time.

**Table 3:** Average of area (mAU*min) for each component detected in O. *ficus-indica* (L.) Mill. samples at 280 nm, except components 14–15 (474 nm), during cold and shelf life storage.

Id.	Component		D0	D7	D14	D21	D28
1	L-Malic acid	After cold storage	7.67 ± 0.86 A ^†^	6.23 ± 0.68 Aa	11.32 ± 2.50 Aa	8.71 ± 2.58 Aa	8.0 ± 2.38 Aa
		After shelf life	7.67 ± 0.86 A ^†^	7.93 ± 1.25 Aa	15.39 ± 2.09 Aa	10.87 ± 3.15 Aa	6.04 ± 3.20 Aa
2	Citric acid	After cold storage	45.3 ± 3.94 B	44.62 ± 2.01 Ba	29.45 ± 4.05 Aa	24.50 ± 4.18 Aa	28.58 ± 6.65 Aa
		After shelf life	45.3 ± 3.94 A	40.40 ± 3.2 Aa	40.18 ± 4.92 Ab	40.21 ± 4.04 Ab	32.49 ± 4.52 Aa
3	Piscidic acid	After cold storage	166.85 ± 25.99 A	172.18 ± 5.59 Aa	161.62 ± 11.07 Aa	158.07 ± 7.99 Aa	173.45 ± 3.33 Aa
		After shelf life	166.85 ± 25.99 A	180.19 ± 17.40 Aa	162.25 ± 4.10 Aa	150.47 ± 16.59 Aa	171.55 ± 26.44 Aa
5	Eucomic acid	After cold storage	43.77 ± 13.25 A	42.10 ± 1.67 Aa	37.81 ± 2.88 Aa	42.66 ± 6.14 Aa	37.66 ± 3.32 Aa
		After shelf life	43.77 ± 13.25 A	47.84 ± 4.71 Aa	42.90 ± 12.43 Aa	30.96 ± 2.81 Aa	40.47 ± 7.38 Aa
7	Eucomic acid isomer/derivative	After cold storage	19.85 ± 1.45 A	24.19 ± 0.97 Ba	25.52 ± 0.54 Ba	24.32 ± 0.73 Ba	25.25 ± 0.89 Ba
		After shelf life	19.85 ± 1.45 A	24.16 ± 1.67 Aa	25.84 ± 1.27 Aa	22.77 ± 3.58 Aa	24.54 ± 2.20 Aa
8	Ferulic acid derivative	After cold storage	50.35 ± 2.97 AB	49.55 ± 2.35 Aa	58.00 ±1.66 Ba	56.66 ± 2.77 ABa	52.49± 2.21 ABa
		After shelf life	50.35 ± 2.97 A	63.56 ± 7.57 Ab	57.95 ± 6.30 Aa	59.43 ± 3.40 Aa	57.81 ± 6.68 Aa
12	Syringaresinol	After cold storage	27.28 ± 1.61 A	31.48 ± 0.37 Ba	33.99 ± 0.67 Ba	31.42 ± 0.71 Ba	33.43 ± 0.83 Ba
		After shelf life	27.28 ± 1.61 A	30.77 ± 2.56 Aa	32.74 ± 0.20 Aa	29.87 ± 4.35 Aa	34.15 ± 3.14 Aa
13	Feruloyl derivative	After cold storage	6.79 ± 2.11 A	5.74 ± 1.20 Aa	8.38 ± 1.97 Aa	8.25 ± 1.03 Aa	6.55 ± 1.27 Aa
		After shelf life	6.79 ± 2.11 A	11.55 ± 1.77 Aa	7.38 ± 1.55 Aa	9.42 ± 2.16 Aa	8.77 ± 2.92 Aa
14–15	Betaxanthin-proline (indicaxanthin) isomers 1 and 2	After cold storage	21.00 ± 1.03 A	25.55 ± 1.62 Aa	23.42 ± 3.13 Aa	23.66 ± 1.01 Aa	23.52 ± 2.14 Aa
		After shelf life	21.00 ± 1.03 A	27.72 ± 4.29 Aa	25.67 ± 1.32 Aa	21.46 ± 2.04 Aa	22.15 ± 1.15 Aa
16	Tryptophan	After cold storage	19.21 ± 6.51 A	20.03 ± 8.77 Aa	18.21 ± 3.21 Aa	14.60 ± 0.53 Aa	12.17 ± 1.03 Aa
		After shelf life	19.21 ± 6.51 A	15.73 ± 1.25 Aa	21.71 ± 2.65 Aa	14.85 ± 3.25 Aa	18.22 ± 2.81 Aa

^†^ Data are the mean ± standard error SE (*n* = 3). The different capital letters within the rows (A, B, AB) indicates significant differences according to the Tukey test (*p* < 0.05) throughout cold and shelf life storage. Lowercase letters within the columns (a, b) indicate significant differences according to the Tukey test (*p* < 0.05) each day independently compared under cold and shelf life conditions.

## Data Availability

Not applicable.

## Supplementary Materials

The following are available online at https://www.mdpi.com/article/10.3390/foods11020160/s1, Table S1: Gradient used in HPLC analysis; Table S2: Gradient used in HPLC-DAD/MS/MS analysis.
