# Supplementary material for: Phytochemical Profile of Opuntia ficus-indica (L.) Mill Fruits (cv. ‘Orito’) Stored at Different Conditions
Source: Foods. 2022 Jan 8;11(2):160. doi: 10.3390/foods11020160 (PMC8774991; doi:10.3390/foods11020160)
Supplement: Supplementary file 1 [file foods-11-00160-s001.zip › foods-1493412-supplementary.pdf]

Table S1: Gradient used in HPLC analysis

**Time (min)**	**%Eluent A (0.5% HCOOH)**	**% Eluent B (90% ACN + 0.5% HCOOH + H2O)**
0.1	94.4	5.6
15	83.3	16.7
20	77.8	22.2
30	77.8	22.2
55	66.7	33.3
80	0	100
85	0	100
105	94.4	5.6
115	94.4	5.6

Table S2: Gradient used in HPLC-DAD/MS/MS analysis

**Time (min)**	**%Eluent A (0.5% HCOOH in H_2O)**	**% Eluent B (ACN)**
0	95	5
15	85	15
20	80	20
30	80	20
55	70	30
80	10	90
85	10	90
105	95	5
115	95	5
